# Hepatic Effects of Etoricoxib in Mice: Integrated Histopathological and Gene Expression Analysis

**DOI:** 10.3390/ph19030414

**Published:** 2026-03-03

**Authors:** Yahya F. Jamous, Badrah S. Alghamdi, Yazun Jarrar, Emad A. Hindi, Mohammad Z. Alam

**Affiliations:** 1 Wellness and Preventive Medicine Institute, Health Sector, King Abdulaziz City for Science and Technology (KACST), Riyadh 11442, Saudi Arabia; 2 Department of Physiology, Neuroscience Unit, Faculty of Medicine, King Abdulaziz University, Jeddah 21589, Saudi Arabia; basalghamdi@kau.edu.sa; 3 Neuroscience and Geroscience Research Unit, King Fahd Medical Research Center, King Abdulaziz University, Jeddah 21589, Saudi Arabia; 4 Department of Basic Medical Sciences, Faculty of Medicine, Al-Balqa Applied University, Al-Salt 19117, Jordan; 5 Department of Clinical Anatomy, Faculty of Medicine, King Abdulaziz University, Jeddah 22254, Saudi Arabia; 6 Department of Medical Laboratory Technology, Faculty of Applied Medical Sciences, King Abdulaziz University, Jeddah 21589, Saudi Arabia

**Keywords:** arachidonic acid metabolism, COX-2 inhibitor, drug-induced liver injury, etoricoxib, hepatotoxicity

## Abstract

**Background**: Etoricoxib, a selective cyclooxygenase-2 (COX-2) inhibitor, is widely prescribed for the management of inflammatory conditions. Despite its extensive clinical use, evidence regarding its hepatic safety profile remains limited and incompletely characterized. **Aims**: This study aimed to systematically evaluate the hepatic effects of etoricoxib in a murine model by integrating histopathological assessment with analysis of mRNA expression of key enzymes involved in arachidonic acid metabolism **Methods**: Male BALB/c mice (*n* = 7 per group) received either low or high doses of etoricoxib (10.5 or 21 mg/kg/day) or celecoxib (35 or 70 mg/kg/day) for 28 consecutive days. Liver tissues were examined histologically using hematoxylin and eosin staining, while molecular alterations were assessed by quantitative PCR targeting representative cyclooxygenase (COX), lipoxygenase (LOX), and cytochrome P450 (CYP450) isoforms involved in arachidonic acid metabolism. **Results**: High-dose etoricoxib exposure was associated with pronounced hepatic histopathological alterations, including hepatocellular necrosis, inflammatory cell infiltration, and sinusoidal congestion. In contrast, low-dose treatment resulted in only mild vascular and cellular changes. At the molecular level, etoricoxib administration was associated with marked downregulation of several arachidonic acid–metabolizing genes (including *Cyp4a12* and *Alox12*), whereas *Cox2* expression was significantly upregulated (*p* < 0.05), indicating a shift toward a pro-inflammatory transcriptional profile. **Conclusions**: Etoricoxib exposure is associated with dose-dependent hepatic injury in mice, accompanied by coordinated transcriptional alterations in arachidonic acid–metabolizing pathways. Notably, molecular changes were detectable even at low doses in the absence of overt histological damage, suggesting potential early indicators of hepatic stress. These findings underscore the importance of cautious dose optimization and further translational studies to clarify the long-term hepatic safety of etoricoxib in clinical settings.

## 1. Introduction

Arachidonic acid, a fatty acid present in membranes, regulates a variety of biochemical and physiological processes. Upon cellular stimulation, arachidonic acid is released from the membrane via enzymatic pathways and subsequently interacts with multiple metabolic enzymes to generate bioactive mediators with distinct biological functions [1]. The three principal enzyme families in the metabolic pathways of arachidonic acid are lipoxygenases (LOXs), cytochrome P450s (CYP450s), and cyclooxygenases (COXs) [2]. Changes in the activity of these enzymes correspond to several disorders, including inflammatory and cardiovascular diseases [3], cancer [4], and even toxicity from certain drugs [5]. For example, cases of doxorubicin-induced cardiotoxicity have been reported to have less active arachidonic acid-metabolizing CYP450s [6].

The liver is a vital organ involved in metabolizing both endogenous and exogenous compounds, as well as mediating key biochemical processes related to carbohydrate and lipid metabolism [7]. Arachidonic acid and its metabolites significantly influence liver function, modulating glucose production, lipid metabolism, and oxidative stress responses. For example, prostaglandins (e.g., PGE2, PGF2α) and leukotrienes (e.g., LTB4) have been reported to enhance hepatic glucose output by promoting glycogenolysis and gluconeogenesis, contributing to insulin resistance and hyperglycemia. In contrast, epoxyeicosatrienoic acids (EETs), derived from the CYP450 pathway, have been shown to improve insulin sensitivity and limit excessive glucose production [8]. Similarly, pro-inflammatory arachidonic acid derivatives like LTB4 and 5-HETE are associated with increased lipogenesis and hepatic fat accumulation, exacerbating conditions such as non-alcoholic fatty liver disease, whereas lipoxins and EETs exert protective effects by enhancing fatty acid oxidation and reducing inflammation [9].

Arachidonic acid–metabolizing cytochrome P450 enzymes are also capable of metabolizing compounds other than arachidonic acid, including a variety of drugs. *CYP2C9* and CYP2C19 are involved in the metabolism of numerous prescribed medications, such as proton pump inhibitors and antiplatelet agents [10]. In addition, CYP2J2 has been reported to metabolize the antiarrhythmic drug amiodarone [11].

Non-steroidal anti-inflammatory drugs (NSAIDs) are widely used for their analgesic, antipyretic, and anti-inflammatory properties. Their mechanism of action involves inhibiting COX enzymes, thereby preventing the conversion of arachidonic acid into prostaglandins, which mediate inflammation, pain, and fever [12]. Commonly prescribed NSAIDs include diclofenac and ibuprofen, with the clinical use of etoricoxib increasing in recent years [13].

Etoricoxib is a selective COX-2 inhibitor prescribed for treating inflammatory conditions such as rheumatoid arthritis. Unlike non-selective NSAIDs (e.g., diclofenac and ibuprofen), it has fewer gastrointestinal side effects due to its minimal inhibition of COX-1 [14]. According to the European Medicines Agency (EMA), the recommended maximum adult dose of etoricoxib is between 30 and 120 mg/day, and its clinical use is approved for short-term treatment depending on the indication (https://www.ema.europa.eu/en/medicines/human/referrals/etoricoxib, assessed on 24 Feburary 2026). Previous experimental studies, including our own, have reported that etoricoxib administration in mice is associated with pathohistological changes in the heart and kidney, accompanied by altered mRNA expression of arachidonic acid-metabolizing enzymes [15].

To date, there is limited research on the effects of etoricoxib on the liver [16], particularly regarding its impact on arachidonic acid-metabolizing enzymes. Accordingly, the present study investigates etoricoxib-associated hepatic histopathological alterations and examines corresponding changes in the mRNA expression of arachidonic acid–metabolizing enzymes in a mouse model.

## 2. Results

### 2.1. Effect of Celecoxib and Etoricoxib on Liver Weight Percentage Relative to Body Weight

One-way analysis of variance (ANOVA) revealed no statistically significant differences in relation to liver weight among the treated groups compared with the control group (Table 1).

### 2.2. Histological Results of the Liver

**Figure 1:**
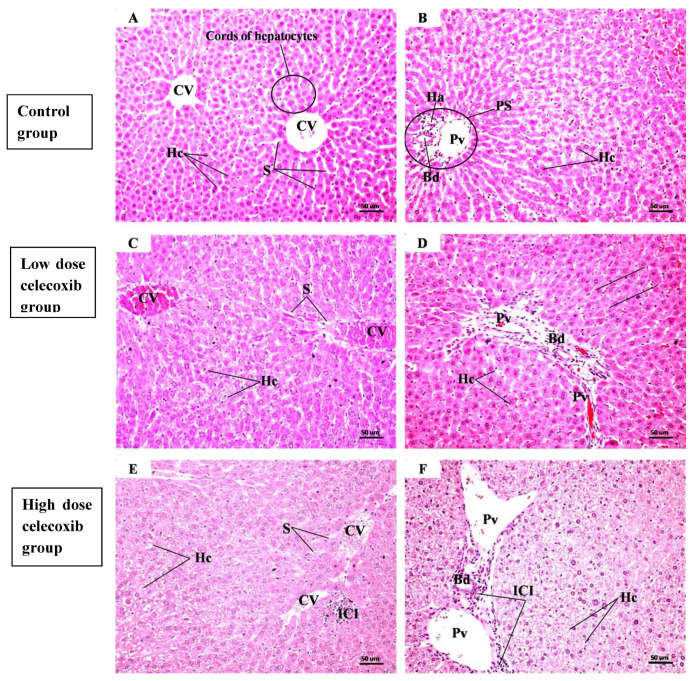

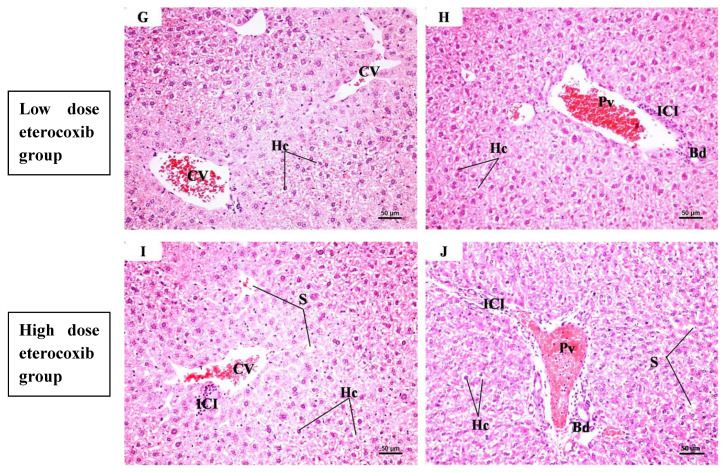
Representative photomicrographs of H&E-stained sections of the centrilobular and periportal regions of the liver lobules from different groups, ×200)**.** Groups: Control group (**A**,**B**), low-dose celecoxib group (**C**,**D**), high-dose celecoxib group (**E**,**F**), low-dose etoricoxib group (**G**,**H**), high-dose etoricoxib group (**I**,**J**). Abbreviations: CV, central vein; PS, portal space; Hc, hepatocytes; S, blood sinusoids; Pv, portal venule; Bd, bile ductule; Ha, hepatic arteriole; ICI, inflammatory cell infiltration. Details about histological alterations in each group are explained in Results.

The histological examinations are illustrated in Figure 1A–J. The control group (A,B) showed preserved hepatic lobular architecture, with a central vein (CV) and radiating cords of hepatocytes separated by blood sinusoids (S). Hepatocytes (Hc) exhibited eosinophilic cytoplasm and large vesicular nuclei with prominent nucleoli. Portal spaces (PS) containing a portal venule (Pv), bile ductule (Bd), and hepatic arteriole (Ha) were observed. The low-dose celecoxib group (C,D) showed mild histological alterations, including dilated and congested central veins and sinusoids, enlarged portal spaces with congested portal venules, bile ductular proliferation, and occasional hepatocellular cytoplasmic vacuolation. The high-dose celecoxib group (E,F) exhibited marked disruption of hepatic lobular architecture, characterized by dilated congested central veins and sinusoids, enlarged portal spaces with bile ductular proliferation, hepatocellular ballooning, cytoplasmic vacuolation, pyknotic nuclei, and inflammatory cell infiltration (ICI). The low-dose etoricoxib group (G,H) demonstrated minimal histological changes, limited to mild dilation and congestion of central veins and sinusoids, with enlarged portal spaces and occasional hepatocyte vacuolation. In contrast, the high-dose etoricoxib group (I,J) showed severe hepatic architectural distortion, with marked vascular congestion, portal expansion with bile ductular proliferation, hepatocyte ballooning with vacuolated cytoplasm and pyknotic nuclei, and prominent inflammatory cell infiltration.

### 2.3. Analysis of mRNA Expression

**Figure 2:**
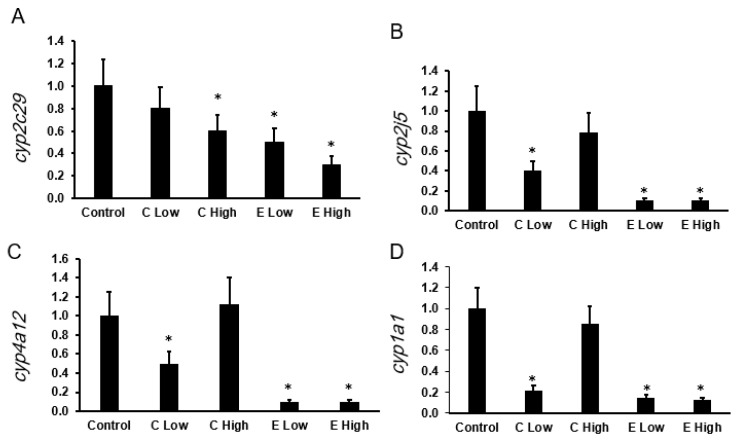
Effect of celecoxib and etoricoxib on the mRNA expression of arachidonic acid–metabolizing CYP450s in liver tissue. The relative mRNA expression levels of (**A**) *Cyp2c29*, (**B**) *Cyp2j5*, (**C**) *Cyp4a12*, and (**D**) *Cyp1a1* were measured by qPCR and are presented as fold change relative to the control group. Mice were treated with low or high doses of celecoxib (C Low, C High) or etoricoxib (E Low, E High) for 28 days. Data are presented as mean ± SD (*n* = 7 mice per group). * indicates a statistically significant difference compared with the control group (*p* < 0.05).

The mRNA expression levels of genes involved in the synthesis of 20-HETE and EETs were expressed as fold changes relative to the control group, as shown in Figure 2A–D. Expression of *Cyp2c29* was significantly reduced in both celecoxib- and etoricoxib-treated groups (Figure 2A). Specifically, high-dose celecoxib significantly decreased *Cyp2c29* expression to approximately 0.65 ± 0.15 (*p* < 0.05), while low and high doses of etoricoxib were associated with further reductions to 0.55 ± 0.13 and 0.35 ± 0.10, respectively (both *p* < 0.05), compared with the control group (1.00 ± 0.25).

The expression of the *Cyp2j5* gene (Figure 2B) was significantly downregulated in the low-dose celecoxib group (0.35 ± 0.15, *p* < 0.05), whereas the high-dose celecoxib group showed a non-significant decrease (0.75 ± 0.20). Notably, both low and high doses of etoricoxib were associated with marked suppression of *Cyp2j5* expression to 0.15 ± 0.08 and 0.10 ± 0.05, respectively (*p* < 0.05).

As shown in Figure 2C, *Cyp4a12*, a key 20-HETE–synthesizing gene, exhibited a significant reduction following low-dose celecoxib treatment (0.50 ± 0.20, *p* < 0.05), whereas high-dose celecoxib did not produce a statistically significant change (1.25 ± 0.35). In contrast, both low and high doses of etoricoxib were associated with pronounced suppression of *Cyp4a12* expression to 0.10 ± 0.05 (*p* < 0.05).

Regarding the expression of *Cyp1a1* (Figure 2D), low-dose celecoxib resulted in a significant decrease (0.30 ± 0.10, *p* < 0.05), while high-dose celecoxib showed a modest, non-significant effect (0.85 ± 0.20). Consistent with the other genes examined, both low and high doses of etoricoxib were associated with significant reductions in *Cyp1a1* expression to 0.25 ± 0.08 and 0.20 ± 0.05, respectively (*p* < 0.05).

**Figure 3:**
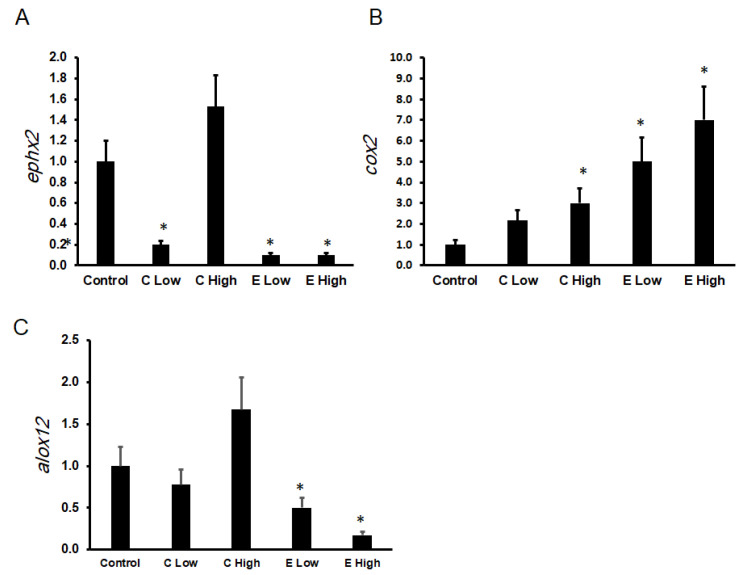
Effect of celecoxib and etoricoxib on the mRNA expression of EET-metabolizing and inflammatory pathway genes. The relative mRNA expression levels of (**A**) *Ephx2*, (**B**) *Cox2*, and (**C**) *Alox12* were quantified by qPCR and expressed as fold change relative to the control group. Mice were treated with low or high doses of celecoxib (C Low, C High) or etoricoxib (E Low, E High) for 28 days. Data are presented as mean ± SD (*n* = 7 mice per group). * indicates a statistically significant difference compared with the control group (*p* < 0.05).

The expression levels of genes involved in the metabolism of EETs and inflammatory mediators, *Ephx2*, *Cox2*, and *Alox12*, were significantly altered following celecoxib and etoricoxib treatment, as shown in Figure 3A–C.

The expression of *Ephx2* was markedly downregulated in all treatment groups except the high-dose celecoxib group (Figure 3A). Low-dose celecoxib significantly decreased *Ephx2* expression to 0.2 ± 0.05 (*p* < 0.05). Both low and high doses of etoricoxib were associated with significant suppression of *Ephx2* expression (0.15 ± 0.04 and 0.1 ± 0.03, respectively; *p* < 0.05).

As illustrated in Figure 3B, *Cox2* expression was significantly increased in all treatment groups compared with the control group. Low-dose celecoxib increased *Cox2* expression to 2.5 ± 0.7, while high-dose celecoxib further increased it to 3.5 ± 1.0 (*p* < 0.05). Etoricoxib treatment resulted in a more pronounced elevation of *Cox2* expression, reaching 6.0 ± 1.2 in the low-dose group and 8.0 ± 1.5 in the high-dose group (*p* < 0.05).

Expression of *Alox12* did not show a statistically significant change in the celecoxib-treated groups compared with the control group (0.9 ± 0.2 in C Low and 1.8 ± 0.5 in C High). In contrast, etoricoxib was associated with a dose-dependent suppression of *Alox12* expression. Low-dose etoricoxib reduced expression to 0.5 ± 0.1 (*p* < 0.05), while high-dose etoricoxib further decreased it to 0.2 ± 0.05 (*p* < 0.05), as shown in Figure 3C.

## 3. Discussion

Etoricoxib is one of the most commonly prescribed anti-inflammatory drugs in many countries [13]. However, its safety profile concerning liver toxicity remains incompletely characterized. This study investigated the effects of etoricoxib on mouse liver by evaluating histopathological alterations associated with its administration and examining corresponding changes in the mRNA expression of arachidonic acid–metabolizing enzymes. The findings provide additional insight into the hepatic safety profile of etoricoxib.

The 28-day treatment period was employed in the animal model, of this study, was designed to evaluate the sub-chronic exposure and does not directly correspond to the approved duration of etoricoxib use in humans, which is typically limited to shorter treatment courses.

Some clinical reports have indicated that etoricoxib may be associated with hepatotoxicity, as evidenced by elevated hepatic enzyme levels and biomarkers of hepatic necrosis [17]. In the present study, histological evaluation revealed hepatocellular necrosis in mice exposed to high doses of etoricoxib. No necrotic changes were observed in the control group or in mice treated with low doses of celecoxib or etoricoxib. Liver tissues from the control group exhibited normal histological architecture, characterized by intact hepatocytes, well-defined nuclei, and organized sinusoidal spaces. Similarly, low-dose treatment groups displayed only minor histopathological changes, such as mild vascular congestion, slight expansion of portal areas, and occasional hepatocellular vacuolization. These findings are indicative of early or mild cellular stress rather than overt tissue injury.

In contrast, pronounced hepatocellular damage and necrosis were observed in mice administered high doses of either drug. These effects included hepatocyte ballooning, nuclear pyknosis, and significant inflammatory cell infiltration around central veins and portal tracts. Collectively, these histopathological features are consistent with drug-associated liver injury at higher doses, supporting the conclusion that etoricoxib is associated with dose-dependent hepatic injury in this experimental model. Accordingly, caution may be warranted when high doses are administered, particularly in individuals with risk factors such as drug–drug interactions or genetic variations in drug-metabolizing enzymes and transporters [18,19]. Additionally, caution is warranted when prescribing high doses to patients with pre-existing hepatic impairment [20].

This study also revealed that the mRNA expression of most arachidonic acid–metabolizing enzyme genes was significantly reduced, with the exception of *Cyp2c9* and *Cox2*. These findings align with previous reports demonstrating decreased expression of arachidonic acid–metabolizing enzymes in the livers of mice treated with metallic nanoparticles [21] or classical NSAIDs [5]. Furthermore, similar reductions have been observed in the livers of uncontrolled diabetic mice [22]. Taken together, these observations suggest that transcriptional downregulation of arachidonic acid–metabolizing enzymes may represent a common molecular feature associated with both drug-related hepatotoxicity and disease-associated liver injury.

Interestingly, *Cox2* expression was consistently upregulated following etoricoxib administration, even at low doses. The COX-2 enzyme metabolizes arachidonic acid into pro-inflammatory prostaglandins [23]. This transcriptional increase parallels the inflammatory cell infiltration observed histologically, particularly following high-dose etoricoxib exposure. While a direct causal relationship cannot be established, the upregulation of *Cox2* may contribute to an enhanced inflammatory milieu within the liver. This observation is consistent with previous studies reporting elevated *Cox2* expression in liver diseases and toxicological conditions [24]. The precise mechanism underlying etoricoxib-associated *Cox2* upregulation remains unclear, but it may occur indirectly through pathological changes that activate inflammatory signaling cascades, subsequently increasing the expression of inflammation-related enzymes [25].

Notably, low-dose etoricoxib induced significant transcriptional changes in arachidonic acid–metabolizing enzymes while producing only minimal histopathological alterations. This apparent discrepancy may reflect the higher sensitivity of molecular endpoints compared with structural tissue changes, which often manifest following prolonged exposure or higher drug concentrations [26]. Previous studies have reported cases in which pharmacological agents caused substantial alterations in hepatic biomarkers or gene expression without corresponding histopathological damage [27,28,29].

Celecoxib was included as a positive control due to its well-documented effects on hepatic function and arachidonic acid metabolism [25,30]. Comparative analysis revealed that etoricoxib and celecoxib elicited broadly similar hepatic responses, with both drugs inducing histopathological alterations at high doses and transcriptional changes at both low and high doses. These findings suggest that the hepatic effects of etoricoxib may be comparable to those of celecoxib under experimental conditions. The pharmacological and toxicological similarities between the two compounds may partly reflect their structural and mechanistic relatedness as selective COX-2 inhibitors.

Although etoricoxib induced histopathological alterations and was associated with transcriptional changes in arachidonic acid–metabolizing genes, the relative liver weight of treated mice remained comparable to that of the control group. Previous studies have reported mixed findings regarding liver weight changes in drug-induced hepatotoxicity, with some demonstrating significant differences [31], while others did not [32,33]. This variability suggests that liver weight alone may not be a sensitive or reliable indicator of drug-induced hepatic injury, underscoring the importance of combined histological and molecular assessments. Recent experimental studies have highlighted similar dysregulation of arachidonic acid pathways following COX-2 inhibition, supporting the concept that selective COX-2 inhibitors may exert off-target hepatic effects through inflammatory and metabolic reprogramming [17].

Lastly, we observed that both low and high doses of etoricoxib induced significant molecular alterations, whereas the low dose showed more pronounced changes compared with the high-dose celecoxib group. These findings suggest that low doses of both celecoxib and etoricoxib are capable of eliciting molecular changes in the expression of hepatic arachidonic acid–metabolizing cytochrome Cyp450 genes. In contrast, the high dose of celecoxib did not demonstrate comparable significant effects, which may be attributed to increased variability within this treatment group, potentially affecting the statistical power and significance of the analysis, or to other mechanisms that remain to be elucidated.

### Strengths and Limitations

A key strength of this study lies in the integrated assessment of hepatic injury, combining detailed histopathological analysis with comprehensive transcriptional profiling of arachidonic acid–metabolizing enzymes. The inclusion of both low and high doses, along with celecoxib as a comparator, enabled evaluation of dose-dependent effects and provided relevant pharmacological context. However, some limitations should be acknowledged. The findings are derived from an animal model and may not fully reflect human hepatic responses. In addition, the study focused on mRNA-level changes and histopathology; functional biochemical markers of liver injury and protein-level validation were not assessed. Although serum biochemical parameters of liver function were measured in the present study, these data were obtained using general assay kits not specifically validated for mouse samples and were therefore not included in the final statistical analysis. Furthermore, not all genes involved in arachidonic acid metabolism were analyzed in this study, including COX-1. Future studies incorporating functional, proteomic, and clinical endpoints will be important to further clarify the translational relevance of these findings.

## 4. Materials and Methods

### 4.1. Chemicals

Celecoxib (purity >99%; Cat. No. C9180) and etoricoxib (purity >98%; Cat. No. E2470) were obtained from Solarbio (Beijing, China). Additional reagents included a total RNA extraction kit (R1200), a universal reverse transcriptase–polymerase chain reaction (RT-PCR) kit (M-MLV, RP1105), and SYBR Green PCR Master Mix (SR1110) (Solarbio, China). PCR primers were synthesized by Integrated DNA Technologies (Coralville, IA, USA).

### 4.2. Experimental Animals

Thirty-five male BALB/c mice (6–8 weeks old, 29 ± 3 g) were procured from the animal facility of King Fahd Medical Research Center (KFMRC). Mice were housed under controlled conditions (12 h light/dark cycle, 22 ± 2 °C, 50–60% humidity) with ad libitum access to standard chow and water. All procedures complied with the guidelines of the KFMRC Animal Care and Use Committee (ACUC protocol no. 22-1-5) and were approved by the Biomedical Ethics Committee of King Abdulaziz University (Approval No. 69-22).

### 4.3. Experimental Design

The experimental protocol was conducted as previously described [15]. Briefly, mice were randomly allocated into five groups (*n* = 7 per group):

Control: Received 0.2 mL/day of vehicle (cellulose) orally (PO).
Low-dose celecoxib (35 mg/kg/day, PO).
High-dose celecoxib (70 mg/kg/day, PO).
Low-dose etoricoxib (10.5 mg/kg/day, PO).
High-dose etoricoxib (21 mg/kg/day, PO).

Based on body surface area–based dose conversion, the murine doses of etoricoxib (10.5 and 21 mg/kg/day) correspond approximately to human therapeutic doses of 60 and 120 mg/day, respectively, while celecoxib doses of 35 and 70 mg/kg/day correspond to human doses of approximately 200 and 400 mg/day. After a 7-day acclimatization period, treatments were administered for 28 consecutive days. Body weight was recorded weekly, and weight gain (%) was calculated as:

Weight gain (%) = [(Final weight − Initial weight)/Initial weight] × 100

At the experimental endpoint, mice were euthanized, and livers were excised, weighed, and processed for histopathological and molecular analyses.

### 4.4. Histopathological Examination

Liver tissues were rinsed in 0.9% saline, fixed in 10% neutral-buffered formalin for 24 h, dehydrated through graded ethanol, cleared in xylene, and embedded in paraffin. Sections (5 µm) were stained with hematoxylin and eosin (H&E) and examined using a Leica® light microscope equipped with a digital camera (Leica Microsystems, Wetzlar, Germany).

Ten randomly selected H&E-stained liver sections (200× magnification) per group were evaluated in a blinded manner using ImageJ software (v1.47, NIH, Bethesda, MD, USA).

RNA Extraction and cDNA Synthesis

Total RNA was isolated from 100 mg of liver tissue using the Solarbio RNA extraction kit (R1200) (Solarbio, Beijing, China). cDNA was synthesized from 1 µg of total RNA using M-MLV reverse transcriptase (RP1105) at 37 °C for 60 min, followed by enzyme inactivation at 65 °C for 5 min.

Quantitative PCR (qPCR) Analysis

The mRNA expression levels of the following genes were analyzed: *Cox2*, *ALox12*, *ALox15*, *Cyp4a12*, *Cyp1a1*, *Cyp2c29*, *Cyp2j5*, and soluble epoxide hydrolase (*Ephx2). β-actin* was used as the reference gene. Primer sequences and amplicon sizes were as previously reported [15]. qPCR conditions consisted of an initial denaturation at 95 °C for 3 min, followed by 40 cycles of denaturation at 95 °C for 10 s and annealing at primer-specific temperatures (53–58 °C) for 30 s. Relative gene expression was calculated using the 2^−^ΔΔCt method [34] and normalized to *β-actin* expression.

### 4.5. Statistical Analysis

Data are expressed as mean ± SEM. Differences between groups were assessed using one-way or two-way analysis of variance (ANOVA), followed by Tukey’s post hoc test SPSS Statistics v26 (IBM Corp., Armonk, NY, USA). A *p*-value < 0.05 was considered statistically significant.

## 5. Conclusions

This study provides integrated histopathological and molecular evidence that etoricoxib induces dose-dependent hepatotoxicity in mice. High-dose exposure resulted in marked hepatocellular injury and inflammatory infiltration, accompanied by profound dysregulation of arachidonic acid–metabolizing enzymes. The suppression of multiple CYP450 and LOX pathways, together with paradoxical upregulation of *Cox2*, suggests a shift toward a pro-inflammatory hepatic environment that may underlie etoricoxib-induced liver injury. Importantly, molecular alterations were detectable even at low doses in the absence of overt histological damage, indicating that changes in arachidonic acid metabolism may represent early biomarkers of hepatotoxicity. Collectively, these findings raise concerns regarding high-dose etoricoxib use and highlight the need for careful hepatic monitoring and further translational studies to assess long-term clinical safety.

## Figures and Tables

**Table 1:** Liver weight percentage relative to body weight.

Groups	Liver (Weight%)
Control	5.152 ± 0.247
Celecoxib (35 mg/kg/day)	5.355 ± 0.125
Celecoxib (70 mg/kg/day)	5.400 ± 0.138
Etoricoxib (10.5 mg/kg/day)	5.184 ± 0.142
Etoricoxib (21 mg/kg/day)	5.289 ± 0.242

Data are presented as mean ± standard error of the mean (SEM).

## Data Availability

The original contributions presented in this study are included in the article. Further inquiries can be directed to the corresponding author.

## Abbreviations

The following abbreviations are used in this manuscript:

AA	Arachidonic acid
COX	Cyclooxygenase
COX-1	Cyclooxygenase-1
COX-2	Cyclooxygenase-2
CYP450	Cytochrome P450
EETs	Epoxyeicosatrienoic acids
H&E	Hematoxylin and eosin
LOX	Lipoxygenase
LTB4	Leukotriene B4
5-HETE	5-Hydroxyeicosatetraenoic acid
NSAID	Non-steroidal anti-inflammatory drug
PGE2	Prostaglandin E2
PGF2α	Prostaglandin F2α
qPCR	Quantitative polymerase chain reaction
